# Twenty-Year Span of Global Coronavirus Research Trends: A Bibliometric Analysis

**DOI:** 10.3390/ijerph17093082

**Published:** 2020-04-28

**Authors:** Yi Zhou, Liyu Chen

**Affiliations:** 1 Xiangya School of Medicine, Central South University, Changsha 410013, China; 2 Department of Microbiology, School of Basic Medical Sciences, Central South University, Changsha 410078, China

**Keywords:** coronavirus, SARS-CoV-2, bibliometrics, SARS, MERS, COVID-19

## Abstract

The coronavirus disease 2019 (COVID-19) pandemic aroused global public concern and became a major medical issue. This study aims to investigate the global research routine and trends of coronavirus over the last twenty years based on the production, hotspots, and frontiers of published articles as well as to provide the global health system with a bibliometric reference. The Web of Science core collection database was retrieved for coronavirus articles published from 1 January 2000 to 17 March 2020. Duplicates and discrete papers were excluded. Analysis parameters including time, regions, impact factors, and citation times were processed through professional software. A total of 9043 coronavirus articles originated from 123 countries and were published in 1202 journals. The USA contributed most articles (3101) followed by China (2230). The research was published in specialized journals including the Journal of Virology. Universities were the main institutions of science progress. High-impact articles covered fields of basic science and clinical medicine. There were two sharp increases in research yields after the severe acute respiratory syndrome (SARS) and the Middle East respiratory syndrome (MERS) outbreaks. International collaborations promoted study progress, and universities and academies act as the main force in coronavirus research. More research on prevention and treatment is needed according to an analysis of term density.

## 1. Introduction

A novel virus named severe acute respiratory syndrome coronavirus 2 (SARS-CoV-2) emerged in Wuhan, China in December 2019, causing a large global outbreak: the coronavirus disease 2019 (COVID-19) pandemic [1,2,3]. It has aroused international public concern and has become a major medical issue [4,5]. Within the past two decades, coronavirus infection has broken out recurrently and triggered three widespread epidemics: The severe acute respiratory syndrome (SARS) occurred in China in 2002 [6], the Middle East respiratory syndrome (MERS) emerged in Saudi Arabia in 2012 [7], and now, COVID-19. Pathogens of the above diseases are species in the genus of *Betacoronavirus* belonging to the family Coronaviridae in the order Nidovirales [8]. Collaborative research from epidemiology and etiology, including identifying pathogens, tracking sources, and clinical prevention and treatment including vaccines and antivirus medicine discovery, are urgent [9,10]. Bibliometrics based on the mapping knowledge domain as a tool to evaluate the research outputs’ characteristics has been widely adopted, and analysis results are capable of providing a comprehensive assessment of the quality and quantity of scientific yields [11,12]. Such research can not only describe the trends and distribution of publications including the impacts and citations but also reflect health policy decisions, the input of medical resources, and further social phenomena [13]. In order to assess the impact of coronavirus research on global scientific research production and contribute to the prevention and control of COVID-19, a bibliometric analysis was performed by utilizing the accessible date indexed at the Web of Science database.

## 2. Materials and Methods

**Figure 1:**
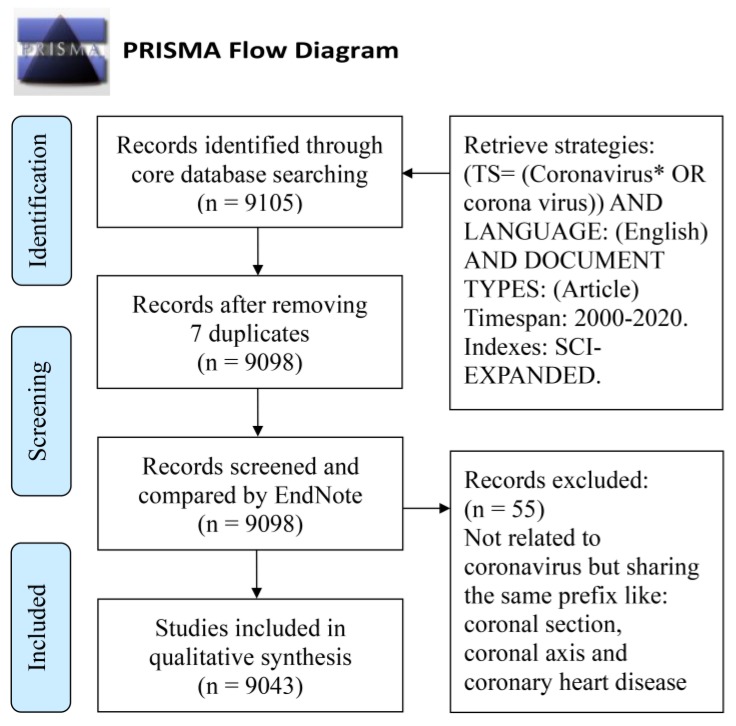
Flowchart of data screening and inclusion.

This bibliometric study analyzed a twenty-year span of publications on coronavirus research from 1 January 2000 to 17 March 2020. The data used for analysis were extracted from the Web of Science Core Collection (WoSCC) bibliographic database. The retrieve strategies were as follows: (TS = (coronavirus* OR corona virus)) AND LANGUAGE: (English) AND DOCUMENT TYPES: (Article). Timespan: 2000–2020. Indexes: SCI-EXPANDED (Figure 1).

The retrieved articles were screened by using the software EndNote X9.6 (Clarivate Corporation, Philadelphia, PA, USA). Duplicates and other types of literature including book chapters and conference records were removed from extraction firstly. Articles whose keywords share the same prefix, such as coronary heart disease or coronal section, but do not focus on coronavirus were excluded as well. Profile information of included articles were processed and transferred to a local EndNote database, including the title, the date of publication, the corresponding authors with addresses, the rest of the authors, the journal name with the impact factor (IF) value, affiliations, and the source country. The processes above were accomplished in two days 19–20 March 2020 to avoid potential bias related to database updates. After that, a Standard Competition Rank (SCR) report of most productive journals, countries or territories, institutions, corresponding authors, and most-cited articles were summarized and produced.

Data analysis was performed using the Statistical Package for Social Sciences (SPSS; Version 19.0. IBM corporation, Armonk, NY, USA). The software CiteSpace R3 Version 5.4 (Chaomei Chen, College of Computing and Informatics, Drexel University, Philadelphia, PA, USA) was used for visualizing international collaborations and internal links of coronavirus research [14]. The threshold was set to 10, and the node size 15. The software VOSviewer (Center for Science and Technology Studies, Leiden University, Leiden, Netherlands) was used for relatively quantifying the frequency and density of coronavirus articles’ core terminologies [15]. Information on the impact factor (IF) value (the average number of citations up to two years after publication) was acquired from the Journal Citation Report (JCR) 2019, Science Edition (Thomson Reuters).

## 3. Results

### 3.1. Original Artcles Reached

According to the refined retrieve strategies, a detailed search for coronavirus publication obtained 9105 articles from Web of Science, in which seven duplications were deleted, leaving 9098 articles. Afterward, 55 articles were removed due to their unmatched key terms after careful screening. Therefore, a total of 9043 articles were included and saved for the next step in the process (Figure 1).

### 3.2. Yearly Yields

**Figure 2:**
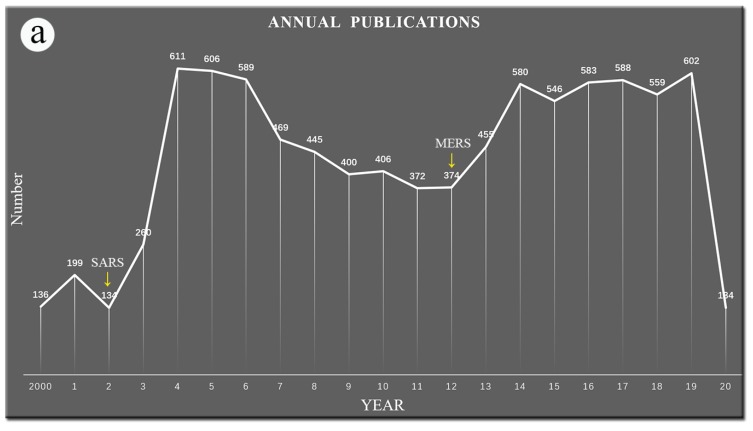

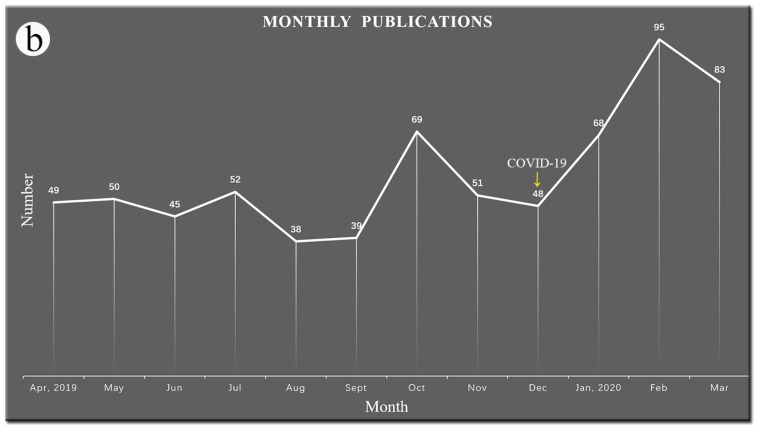
The time-related publications variation. Yellow arrows direct the key time points of SARS (severe acute respiratory syndrome), MERS (Middle East respiratory syndrome), and COVID-19 (coronavirus disease 2019) outbreaks. (**a**) The number of annual publications from 2000 to 2020; (**b**) the monthly publications from April 2019 to March 2020.

Among these articles, 611 of them were published in 2004, accounting for 6.757%, the highest percentage, while 136 were published in 2000, accounting for 1.504%, the lowest. Only 235 articles were published in the first two years. After SARS and MERS broke out (yellow arrows shown in Figure 2a), there were two obvious publication bursts two years later. There was a sharp increase two months after the outbreak of COVID-19 as well (Figure 2b).

### 3.3. An Order of Productive Regions

The retrieved articles were from 123 countries, of which the USA ranked first, followed by China, Germany, and Netherlands (Table 1). China is the country where SARS and COVID-19 broke out and severely struck. Saudi Arabia, ranked 12th, is the country where MERS emerged and attacked. Among the 10 most productive regions, China is the only developing one.

### 3.4. The Visualization of Collaboration

**Figure 3:**
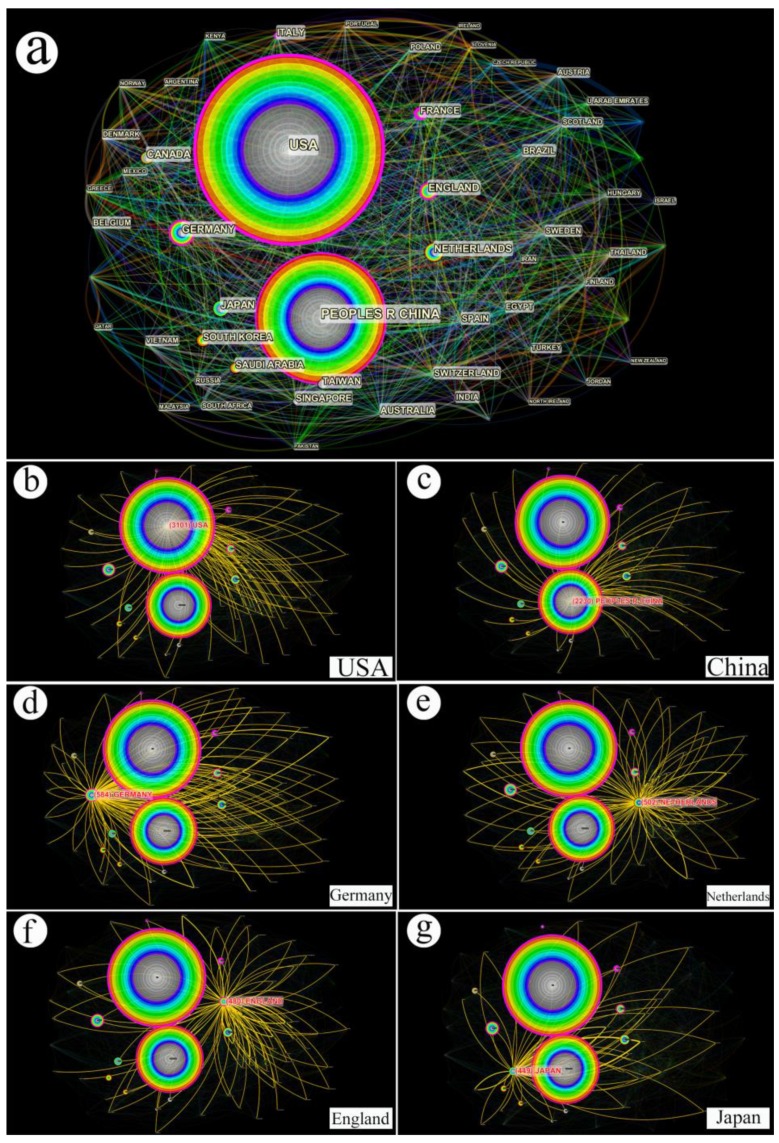
The visualization of international collaboration and internal links on coronavirus research. (**a**) The comprehensive international collaboration network; (**b**) internal links of the USA with other regions involved in 3101 articles; (**c**) internal links of China with other regions involved in 2230 articles; (**d**) internal links of Germany with other regions involved in 584 articles; (**e**) internal links of Netherlands with other regions involved in 502 articles; (**f**) internal links of England with other regions involved in 480 articles; (**g**) internal links of Japan with other regions involved in 449 articles.

The international collaboration of coronavirus research was analyzed and is visualized here (Figure 3a). The diameter of rainbow circles represents the number of cooperative articles, and the differently colored layers of circles indicate the time order of publications. The outer layer in red indicates the recent data, while the inner layer in white is relatively old. The colors of lines obey the same principles. Hence, the USA and China ranked first and second in the two large circles. The top six productive countries were isolated and emphasized from the comprehensive network. The yellow lines indicate the internal links between center and remote spots (Figure 3b–g).

### 3.5. An Order of Productive Journals

Articles of inclusion were published in 1202 different journals, of which the *Journal of Virology* ranked first (883 articles, 9.764%), followed by *Virology* (285 articles, 3.152%), and *PLoS ONE* (242 articles, 2.676%), shown in Table 2. The *Journal of Virology*, established by the American Society for Microbiology (ASM), is one of the top journals in the field of virus research. Most of these journals focus on discovering vaccines and antivirus agents, and have relatively high IF values, such as *PNAS* (IF = 9.580) and *Emerging Infectious Diseases* (IF = 7.185).

### 3.6. An Order of Productive Institutions

The contributions of the 15 most productive institutions are ranked in Table 3. University of Hong Kong, the publication number of which is 434, ranked first, accounting for 4.578%, followed by the Chinese Academy of Science, 329 (3.638%), and the University of California System, 246 (2.720%). Among these institutions, nine are from the USA, with four from China and one from Netherlands.

### 3.7. A List of Frequently-Cited Articles

The 10 most cited articles in the field of coronavirus are shown in Table 4. The most frequently cited article was “A novel coronavirus associated with severe acute respiratory syndrome” by Ksiazek et al., published in 2003 in the *New England Journal of Medicine*, followed by “Identification of a novel coronavirus in patients with severe acute respiratory syndrome” by Drosten et al., published at the same time and in the same journal [16,17]. Both of them are cited more than 1700 times. All of these top articles were published in high-impact journals and share an average citation number of 1265.

### 3.8. Terms Analysis and Mapping

**Figure 4:**
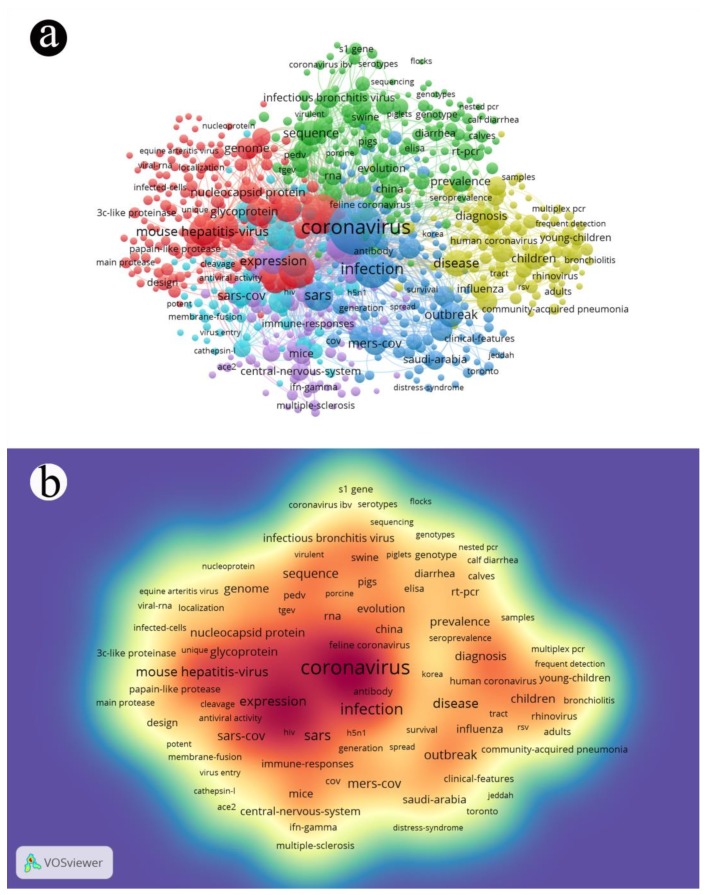
The visualization of keywords or terms used in coronavirus articles. (**a**) Different clusters and internal links between different terms contained in the sphere and lines; (**b**) density map of frequent terms indexed in Web of Science.

In order to analyze keywords or topic terms used in retrieved articles, VOSviewer (Center for Science and Technology Studies, Leiden University, Leiden, Netherlands) as a conventional application of mapping knowledge domain was employed to explore co-cited links and usage frequency. The threshold of term usage was set to 10 before processing. Different clusters are shown in colorful spheres and lines (Figure 4a), while a density map exhibits the research areas (Figure 4b). The front size and color depth of a term reflect its usage density and connections [26]. In Figure 4a, the terms “coronavirus”, “MERS-CoV”, “SARS”, and “SARS-CoV”, which belong to the blue cluster, interact with the terms “prevalence” and “evolution” in the green cluster, “disease” and “diagnosis” in the yellow cluster, “mice” and “central nervous system” in the purple cluster, and “mouse-hepatitis-virus” and “expression” in the red cluster. In Figure 4b, the density of the term “coronavirus” is the highest, followed by “infection”, “SARS”, and “expression”, which reflect the using frequency in articles. The terms “vaccine” and “antiviral agents” are of low frequency.

Guided by cluster and density map combination, articles containing the above terms were extracted from the local database for exploration of the research hotspots and frontiers. Over the past two decades, research focused on coronavirus mainly includes five clusters. (1) The core cluster is the blue one, and concentrates on the biological and virologic characteristics of coronavirus, including essential factors of infection and transmission routes during the outbreaks of SARS and MERS, as well as clinical features. Infection probably spreads through an air-borne route and through close contact. Cases of infection could involve symptoms such as fever, cough, and consolidation of the lung [27]. (2) The red cluster: Some types of coronavirus spread among animals and humans. Laboratory contamination and particularly animal-to-human transmission could be brought by mouse hepatitis-virus (MHV), a kind of coronavirus that can cause infection of the central nervous system and suppress the immune system by influencing immunoglobulin excretion from the B cells of Peyer’s Patch, some immune responses of which resemble pneumonia-associated coronavirus [28]. (3) The yellow cluster: Primary infection of coronavirus in mammals and birds is confined to the upper respiratory and gastrointestinal system. Seven different known strains of coronaviruses are capable of infecting humans, in which SARS-CoV, as a publicized human coronavirus, has a unique pathogenesis because it causes both upper and lower respiratory tract infections involving bronchiolitis and pneumonia [29]. (4) The purple cluster: The entrance into human body of SARS-CoV depends on the angiotensin-converting enzyme 2 (ACE2) receptor, while the spike protein functions as the adaptor. Interferon-gamma participate in the immune-response acting as an antiviral agent. Medicine could be discovered according to these features [30,31]. (5) The green cluster: The evolution based on the mutation of coronavirus RNA caused different symptoms to human kind. High-fidelity whole genome sequencing could be a detection method of mutation besides diagnosis of infection. Population of coronavirus epidemics is mainly young children and the elders, who are in the status of hypo responsiveness [32,33].

## 4. Discussion

SARS-CoV-2 infection was first reported in December 2019, and the infection spread and situation worsened and became a Public Health Emergency of International Concern soon thereafter [34]. Coronavirus infections, including SARS, MERS, and COVID-19, have repeatedly broken out over the last 20 years. To the best of our knowledge, it is the first study to bibliometrically assess the yields of a twenty-year span of publications on coronavirus. Scientific research cannot be considered to promote the advancement of knowledge unless they are published through peer review and editor checks [35]. Our analysis included a total of 9043 original articles on coronavirus that were published over the past two decades, and this amount was enough to relatively reflect the scientific trends and development and certain social phenomena. Our analysis showed that coronavirus research articles were from multiple countries, from which numerous scientists have participated in the defense against coronavirus. The two outbursts of literature immediately after the outbreak of SARS and MERS indicate that response was quick employed and great importance was immediately attached in life sciences, basic medicine, and clinical pharmacy.

The USA and China are the most productive countries, contributing over 5400 articles to coronavirus research. Saudi Arabia and South Korea also ranked high at 13th and 8th places, respectively. These results are not surprising because the USA has been crucial in fostering and engaging in international collaborations on coronavirus research regarding prevention, control, diagnosis, and treatment concerning the possible risk of a global spread due to the strength of its economic implications. Furthermore, China, Saudi Arabia, and South Korea have been seriously attacked by coronavirus, and great effort has been made in basic science and clinical medicine. Internal links forming a comprehensive and extensive network indicate that research collaborations between nations and continents have been extensively carried out. Cooperation as a conventional development modality, which sharing resources, authorities, communication, and even sensation has become a trend in global research [36]. Articles from around the world have been checked, peer-viewed, and eventually published in journals and delivered all over the world. Articles were accepted mainly from specialized journals in the fields of virology and microbiology with high impact factors such as the Journal of Virology, Emerging Infectious Diseases, the Journal of Clinical Microbiology, and PNAS. Information and knowledge as vectors to load experiments and research advances have been found across nations and regions, leading to solutions and progress [37].

Institutions can be classified into several categories: associated government departments, academies, research institutes, universities and affiliated hospitals, the Center for Disease Control and Prevention (CDC), and others. Among the 15 most productive institutions, universities account for more than half of the research output, followed by academies, government departments, and the CDC, indicating them as the most dynamic and creative. However, the most productive institution has been the University of Hong Kong. This might be ascribed to tremendous losses due to the outbreak of SARS in situ and the easier access to data and specimens [38].

It is apparent that the IF value could be an efficient but relatively controversial tool to quantitively evaluate the performance and influence of a peer-review journal. Not being an absolute criterion of quality, IF as an article citation measurement still reflects the impact that indexed journals have under certain disciplines [39]. The 10 most cited articles on coronavirus were all published on the eminent journals with a high IF including the *New England of Medicine, Science, Lancet*, and *Nature.* Reports of the first case of SARS and MERS were cited the most frequently. Clinical studies including characterization and transmission, virologic studies including identification and isolation of pathogen, and molecular studies including genome sequence and infection mechanism have also been cited multiple times [16,17,18,19,20,21,22,23,24,25].

Basic science and clinical trials have been widely carried out, and some progress has been made. However, licensed vaccines and medicine to prevent coronavirus infection has still not been discovered, while choices and formulas of clinical treatment are of limitation. Some potential therapies have been put forward by doctors, including the Lopinavir-Ritonavir combination. A trial for hospitalized adults with severe COVID-19 has recently been declared a failure [40]. Preventive methods mainly aim to reduce the chance of getting infected such as cutting transmission routes by disinfection and sterilization, protecting susceptible populations by allocating the wearing of masks, and effective vaccine injections. It is noteworthy that the COVID-19 pandemic has triggered a momentous milestone in coronavirus research, and extensive international cooperation is tackling this situation with quick reaction. Open access to the whole genome sequence group, disease predictive model construction, and the shared service of the nation health database has accelerated the research targeting the control of the outbreak. Further research efforts in prevention and treatment and in the translation of research creation and new findings into valid clinical measures are both urgent [41].

Limitations exist in this study. First, the Web of Science core collection database is the only retrieval source, and it does not index all journals, so articles from other databases such as Scopus and PubMed might have been ignored [42]. Second, bias might also occur as a result of retrieving from a database without the use of other languages, for instance, Chinese and Arabic, the official language in China and Saudi Arabia, in which many cases were published. Third, the latest articles which have been accepted but not published were not included in this study due to the information delay. While these limitations are important, they are not likely to have caused the patterns and trends that we observed in the data. We believe that these patterns and trends are conclusive, despite these limitations.

## 5. Conclusions

A twenty-year-span of coronavirus research outputs was investigated and analyzed through bibliometric methods based on Web of Science. There were two sharp increases in research yield after the SARS and MERS outbreaks. This research demonstrates that international collaborations have promoted study progress, and universities and academies have acted as the main force participating in basic science and clinical medicine research. High-impact articles, which cover biological features including the genome group, the virulence range, and combine receptors, as well as the pathogenic characteristics including transmission routes, atypical symptoms, and immune response, were published in specialized journals. However, terms such as vaccine and prescription were identified with a low frequency, which indicates that more prevention and treatment research is needed. These findings provide a relatively objective reference for peer scientists, national regimes, and the global health system.

## Figures and Tables

**Table 1:** The 15 most productive countries or territories contributed to articles on coronavirus research.

SCR ^1^	Country/Territory	Number	Percentages
1st	USA	3101	34.292%
2nd	PR China	2230	24.660%
3rd	Germany	584	6.458%
4th	Netherlands	502	5.551%
5th	England	480	5.308%
6th	Japan	449	4.965%
7th	Canada	445	4.920%
8th	South Korea	392	4.335%
9th	China Taiwan	361	3.992%
10th	France	348	3.848%
11th	Italy	313	3.461%
12th	Saudi Arabia	291	3.218%
13th	Singapore	274	3.030%
14th	Australia	267	2.953%
15th	Spain	231	2.554%

^1^ SCR: standard competition ranking. Equal members have the same ranking number, and a gap is left in the ranking numbers.

**Table 2:** The 15 most productive journals with coronavirus articles.

SCR ^1^	Journal Names	Number	Percentages
1st	*Journal of* *Virology*	883	9.764%
2nd	*Virology*	285	3.152%
3rd	*PLoS ONE*	242	2.676%
4th	*Emerging Infectious Diseases*	204	2.256%
5th	*Journal of General Virology*	188	2.079%
6th	*Virus Research*	175	1.935%
7th	*Advances in Experimental Medicine and Biology*	169	1.869%
8th	*Archives of Virology*	155	1.714%
9th	*Journal of Virological Methods*	150	1.659%
10th	*Veterinary Microbiology*	145	1.603%
11th	*Journal of Medical Virology*	119	1.316%
12th	*Journal of Clinical Microbiology*	112	1.239%
13th	*PNAS*	108	1.194%
13th	*Viruses-Basel*	108	1.194%
15th	*Vaccine*	99	1.095%

^1^ SCR: standard competition ranking. Equal members have the same ranking number, and a gap is left in the ranking numbers.

**Table 3:** The 15 most productive institutions that contributed to articles on coronavirus research.

SCR ^1^	Institution	Number	Percentages
1st	University of Hong Kong, China	434	4.578%
2nd	Chinese Academy of Science	329	3.638%
3rd	University of California System, USA	246	2.720%
4th	National Institutes of Health, USA	240	2.654%
5th	Center for Disease Control and Prevent, USA	212	2.344%
6th	University of North Carolina, USA	209	2.311%
7th	Utrecht University, Netherlands	201	2.223%
8th	Univ N Carolina Chapel Hill, USA	172	1.902%
9th	Chinese Academy of Agricultural Sciences	166	1.836%
10th	Chinese University of Hong Kong	164	1.814%
11th	National Institute of Allergy Infectious Disease, USA	155	1.714%
12th	University of Iowa, USA	140	1.548%
13th	University of Texas System, USA	139	1.537%
14th	University of Pennsylvania, USA	137	1.515%
15th	Peking Union Medical College, China	133	1.471%

^1^ SCR: standard competition ranking. Equal members have the same ranking number, and then a gap is left in the ranking numbers.

**Table 4:** The 10 most cited articles on coronavirus research.

SCR ^1^	Article Title	Authors	Journal	Times Cited	Date of Publication	IF ^2^
1	A novel coronavirus associated with severe acute respiratory syndrome	Ksiazek, T.G.; Erdman, D; et al. [16]	*New England Journal of Medicine*	1839	15 May 2003	70.670
2	Identification of a novel coronavirus in patients with severe acute respiratory syndrome	Drosten, C.; Gunther, S; Preiser, W; et al. [17]	*New England Journal of Medicine*	1748	15 May 2003	70.670
3	Characterization of a novel coronavirus associated with severe acute respiratory syndrome	Rota, P.A.; Oberste, M.S.; et al. [18]	*Science*	1489	30 May 2003	41.037
4	Coronavirus as a possible cause of severe acute respiratory syndrome	Peiris, J.S.M.; Lai, ST.; et al. [19]	*Lancet*	1444	19 April 2003	59.102
5	Isolation of a Novel Coronavirus from a Man with Pneumonia in Saudi Arabia	Zaki, Ali Moh; van Boheemen, Sander; et al. [20]	*New England Journal of Medicine*	1298	8 November 2012	70.670
6	The genome sequence of the SARS-associated coronavirus	Marra, M.A.; Jones, S.J.M.; et al. [21]	*Science*	1275	30 May 2003	41.037
7	Clinical progression and viral load in a community outbreak of coronavirus-associated SARS pneumonia: a prospective study	Peiris, J.S.M.; Chu, C.M.; Cheng, V.C.C.; et al. [22]	*Lancet*	834	24 May 2003	59.102
8	Angiotensin-converting enzyme 2 is a functional receptor for the SARS coronavirus	Li, W.H.; Moore, M.J.; Vasilieva, N.; et al. [23]	*Nature*	972	27 November 2003	43.070
9	Isolation and characterization of viruses related to the SARS coronavirus from animals in Southern China	Guan, Y.; Zheng, B.J.; He, Y.Q.; et al. [24]	*Science*	897	10 October 2003	41.037
10	Bats are natural reservoirs of SARS-like coronaviruses	Li, W.D.; Shi, Z.L.; et al. [25]	*Science*	857	28 October 2005	41.037

^1^ SCR: standard competition ranking. Equal members have the same ranking number, and a gap is then left in the ranking numbers. ^2^ IF: impact factor. The impact factor value was reported according to the Thomson Reuter Journal Citation Reports (JCR) 2019.
